# Elastase inhibition by natural flavonoids: mechanistic insights and potential therapeutic applications

**DOI:** 10.3389/fnut.2025.1693869

**Published:** 2025-11-06

**Authors:** Lihao Lin, Hongliu Yao, Jinfeng Fu, Wenhui Zhang, Yongxue Li, Yan Wang, Haoyu Shen, Jingang Mo, Yi Guan

**Affiliations:** 1 Department of Neurosurgery, First Hospital of Jilin University, Changchun, China; 2 Department of Natural Organic Chemistry, College of Chemistry, Changchun Normal University, Changchun, China; 3 School of Life Science, Changchun Normal University, Changchun, China

**Keywords:** elastase, natural flavonoid, protein-ligand interaction, conformational change, biological macromolecule, spectroscopic method, molecular docking

## Abstract

Overproduction of elastase plays an important role in the progression of inflammatory diseases. In this study, we compared the inhibitory effects of structurally similar bioactive flavonoids (quercetin, hyperoside, luteolin, and luteoloside) on elastase activity and elucidated their mechanisms of action. Enzyme inhibition assays and fluorescence, ultraviolet–visible (UV–vis), Fourier transform infrared (FT-IR), and circular dichroism (CD) spectroscopy examinations assessed the interactions among flavonoids, elastase, and elastase conformational changes. Molecular docking analyzed binding interactions. Thermodynamic parameters were calculated to determine the forces that stabilize the flavonoid-elastase complexes. Luteolin strongly inhibited elastase, followed by hyperoside, quercetin, and luteoloside. Fluorescence spectroscopy revealed static quenching of all flavonoids, with binding distances indicating non-radiative energy transfer between the flavonoids and elastase. Thermodynamic analysis revealed that hydrogen bonds and van der Waals forces primarily stabilized hyperoside and luteolin, whereas electrostatic interactions stabilized quercetin and luteoloside. UV–vis, FT-IR, and CD spectroscopy confirmed that flavonoids induced conformational changes in elastase, and increased random coil content was correlated with inhibitory strength. Molecular docking results supported these findings, with strong binding affinities between flavonoids and elastase, particularly luteolin and hyperosides. The four natural flavonoids inhibited elastase by altering their secondary structures. Modifications at positions 3 (C-ring) and 7 (A-ring) of flavonoids can enhance elastase inhibition. These findings provide a scientific basis for the development of flavonoid-based anti-inflammatory therapies targeting elastase-related diseases.

## 1 Introduction

Elastase is a highly potent serine protease and one of the most destructive enzymes known. Hydrolyases are characterized by the decomposition of insoluble elastin (1, 2). Elastin is a crucial component of the lungs, blood vessel walls, and other organs, and is an important component of proteins, including fibronectin, laminin, and collagen (3, 4). However, excessive breakdown of elastin and other peptides by elastase results in pathological changes. Elastase is involved in several common diseases, including emphysema, chronic bronchitis, hepatitis, rheumatoid arthritis, and various cardiovascular and cerebrovascular diseases (5–7). Elastase directly affected inflammation occurrence and development. Therefore, it is considered an effective anti-inflammatory target (8).

Anti-inflammatory drugs are the second most widely used category of clinical drugs after anti-infectives. Sivelestat sodium is a synthetic drug developed by ONO Pharmaceutical for the treatment of acute lung injury (9, 10). It is the most effective elastase inhibitor used in clinical practice. However, sivelestat is expensive, causes numerous side effects, and has a limited long-term safety profile (11). Therefore, cheaper and safer elastase inhibitors are needed. Although various synthetic inhibitors have been explored, natural products, particularly flavonoids, are gaining attention owing to their dual anti-inflammatory and enzymatic inhibitory activities. Compared to other natural inhibitors derived from traditional Chinese medicines, such as alkaloids and saponins, flavonoids have higher bioavailability and reduced toxicity at effective doses (12, 13). Flavonoids are natural compounds with diverse bioactivities that inhibit elastase via specific molecular interactions. These include hydrogen bonds, van der Waals interactions, and electrostatic forces, which collectively stabilize flavonoid binding within the enzyme’s active site.

**Figure 1:**
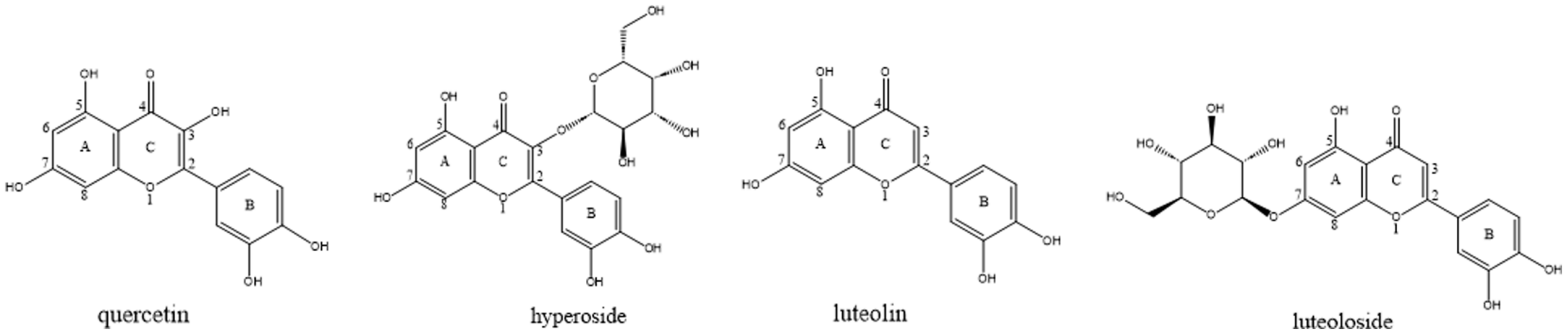
The structures of quercetin, hyperoside, luteolin and luteoloside.

Traditional Chinese medicines (TCMs) have long been considered to have fewer toxic effects and are safer than conventional pharmaceutical drugs (6). Among these natural products, flavonoids represent a particularly promising class due to their dual anti-inflammatory and enzyme inhibitory effects. Previous studies have shown that flavonoids exhibit potent elastase inhibitory effects. However, the mechanisms underlying this activity remain largely unexplored. To ensure the relevance and currency of this information, we focused on recent studies to capture the latest advancements in elastase inhibition and flavonoid-based therapies. Numerous studies have demonstrated that various flavonoids significantly inhibit elastase (14, 15). To identify inhibitors with the above effects, we screened several plant flavonoids with similar structures to inhibit elastase activity. Quercetin, hyperoside, luteolin, and luteoloside (Figure 1) are common, inexpensive, and readily available flavonoids. These drugs exhibit various pharmacological activities, including anti-inflammatory, antioxidant, cardiovascular, and cerebrovascular protection (16–19). We evaluated the inhibitory ability of each monomer on elastase, investigated the interaction mechanism between each monomer and elastase, and determined the structure–activity relationship.

Given the complexity of TCM compositions, characterized by their multi-target and multi-effect properties, and the unclear identification of active ingredients, the development of TCM and studies on their active compounds have largely focused on pharmacodynamic evaluations. However, more systematic investigations of the active ingredients and their mechanisms of action are required. Existing research on natural elastase inhibitors either relies on crude plant extracts without identifying the active monomers, or pharmacodynamic evaluation of the monomers’ activity without exploring their mechanisms of action in detail. Moreover, there has been limited discussion on the inhibitory activity of these compounds and the relationship between their structure and function.

Here, we utilized advanced techniques that included multiple spectroscopy methods and molecular docking to investigate the mechanisms underlying the inhibition of elastase by flavonoids. This study also focuses on how flavonoid binding induces structural changes in elastase, highlighting the molecular recognition and conformational dynamics of this macromolecule.

The findings will inform advancements in biomedical applications, particularly in the development of novel anti-inflammatory therapies. The identification of flavonoids as potent elastase inhibitors opens a promising avenue for treating elastase-related diseases. Furthermore, the molecular insights gained from this study may facilitate the design of more potent and selective inhibitors that could potentially lead to the development of novel therapeutic agents. By targeting elastase inhibition using flavonoids, this study contributes to a growing body of evidence supporting the use of natural products in the treatment of inflammatory diseases.

## 2 Materials and methods

### 2.1 Reagents

High purity (≥98%) quercetin, hyperoside, luteolin, and luteoloside were purchased from Shanghai Acmec Biochemical Co., Ltd. (Shanghai, China). The elastase was purchased from Sigma-Aldrich (St. Louis, MO, United States). Phosphate buffer solution (pH 8.0) was used for circular dichroism (CD) and Fourier transform infrared (FT-IR) spectroscopy. Tris–HCl (pH 7.6) was used as the buffer for all other experiments. All other chemicals were of analytical grade. The flavonoids were dissolved in dimethyl sulfoxide (DMSO) to prepare stock solutions. The final concentration of DMSO in the reaction mixture was maintained below 0.1% (v/v) to avoid any potential interference with porcine pancreatic elastase (PPE) activity or its structural integrity. Previous studies demonstrated that low concentrations of DMSO do not affect the enzymatic activity or conformational stability of serine proteases.

### 2.2 Inhibition ability measurements

Elastase was selected as the enzyme target and MeOSuc-Ala-Ala-Pro-Val-AMC as the substrate for the enzymatic reaction. According to the principle of enzyme reactions, an *in vitro* target enzyme drug screening model was established by measuring the relative fluorescence intensity for drug screening and evaluation. The inhibition rate was measured using a FlexStation3 multifunctional plate reader (Molecular Devices, Shanghai, China). Elastase solution (50 μL, 5 × 10^−7^ mol/L) was mixed with a fluorescent substrate (50 μL, 2 × 10^−5^ mol/L), and Tris–HCl buffer solution was added to obtain a total volume of 200 μL. The mixture was incubated at 298 K for 15 min with shaking for 300 s. The excitation wavelength was adjusted to 380 nm, and the emission wavelength was recorded in the range 420–540 nm. A 200 μL buffer solution was used as the blank for reference. The relative fluorescence values of pure enzyme activity were recorded. Four monomer solutions of the same concentration were added separately and their relative fluorescence values were measured under the same conditions. The concentration of each monomer was varied in different samples to obtain IC_50_ values. Inhibition rate (%) was calculated as follows:


$$\text{Inhibition rate}(\%)=\frac{\mathrm{RF}U_{\mathrm{PPE}}-\mathrm{RF}U_{\text{Drugs}+\mathrm{PPE}}}{\mathrm{RF}U_{\mathrm{PPE}}}\times 100\%$$ (1)

where the relative fluorescence unit (RFU) _PPE_ refers to the relative fluorescence measured when only PPE and the fluorescent substrate are present without any inhibitors, and RFU _Drugs+PPE_ refers to the relative fluorescence measured when both PPE and the test drug inhibitor are present.

### 2.3 Fluorescence spectroscopy measurements

A series of PPE-quercetin, PPE-hyperoside, PPE-luteolin, and PPE-luteoloside solutions were prepared with the concentration of the four monomers as 0, 10, 30, 40, 50, 70, and 80 μM and a PPE concentration of 2 μM and set aside. Blanks for the five sets of experiments were quercetin (80 μM), hyperoside (80 μM), luteolin (80 μM), and luteoloside (80 μM) in the form of quercetin without PPE. Fluorescence measurements were performed using a model F-7000 fluorescence spectrometer (Hitachi, Tokyo, Japan). Elastase solution (2,000 μL in Tris–HCl buffer solution) was mixed with inhibitor solutions at different concentrations in a 1.0 cm quartz cell at 298, 303, and 310 K. The final concentrations of the four natural flavonoids and elastase were identical to those used to obtain the synchronous fluorescence spectra. The fluorescence emission spectra were recorded within the range of 285–500 nm, following excitation at 280 nm. The excitation and emission slit widths were set as 2.5 nm. The scan speed was 1,200 nm/min and the photomultiplier tube (PMT) voltage was 630 V. Fluorescence intensities were corrected for inner filter and dilution effects before analyzing the binding and quenching data.

To determine the type of fluorescence quenching of the monomer interacting with PPE, the Stern-Volmer equation was used (20–22):


$$F_{0}/F=K_{\mathrm{SV}}[Q]+1=K_{q}\tau_{0}[Q]+1$$ (2)

where *F_0_* and *F* are the fluorescence intensities of PPE before and after the addition of different monomer solutions, respectively, *K_sv_* is the quenching constant, [Q] is the concentration of different drugs, *K_q_* is the quenching rate constant, *τ_0_* is the average fluorescence lifetime of the substance in the absence of a quencher, and *τ_0_* for the general biological macromolecule is 1 × 10^−8^ s (20, 23, 24).

### 2.4 Binding constants and binding sites

According to the correction formula, the binding constant (*K_a_*) and number of binding points (*n*) for the actions of the four monomers and PPE were calculated as follows (25–27):


$$\mathrm{lg}\frac{F_{0}-F}{F}={\text{nlgK}}_{a}-\mathrm{nlg}\frac{1}{[Q_{0}]-[P_{0}]\frac{F_{0}-F}{F_{0}}}$$ (3)

where [$Q_{0}$] and [*P_0_*] are the drug and PPE concentrations, respectively.

### 2.5 Thermodynamic parameters and types of forces

Electrostatic, hydrophobic, van der Waals, and hydrogen (H)-bonds exist between PPE and the inhibitors, and their binding forces can be calculated using the van’t Hoff equation (28–30):


$$\begin{array}{c} \ln(K_{2}/K_{1})=\mathrm{\Delta H}(1/T_{1}-1/T_{2})/R \end{array}$$ (4)

$$\begin{array}{c} \ln K=-\mathrm{\Delta H}/\mathrm{RT}+\mathrm{\Delta S}/R \end{array}$$ (5)

$$\begin{array}{c} \mathrm{\Delta G}=\Delta H-\mathrm{T\Delta S}=-\text{RTlnK} \end{array}$$ (6)

where *K* is the binding constant of the interaction, *R* is the gas constant, Δ*H*, Δ*G*, and Δ*S* are enthalpy, Gibbs energy, and entropy change.

### 2.6 Energy transfer and binding distance

The PPE-quercetin, PPE-hyperoside, PPE-luteolin, and PPE-luteoloside binding distances were calculated according to the fluorescence resonance energy transfer theory (31–33):


$$\begin{array}{c} E=1-F/F_{0}=R_{0}^{6}/(R_{0}^{6}+r_{0}^{6}) \end{array}$$ (7)

$$\begin{array}{c} R_{0}^{6}=8.8\times 10^{-25}(k^{2}\varphi n^{-4}J) \end{array}$$ (8)

$$\begin{array}{c} J=\sum F_{D}(\lambda )\varepsilon_{A}(\lambda )\lambda^{4}\Delta \lambda /\sum F_{D}(\lambda )\Delta \lambda \end{array}$$ (9)

where *k*^2^ is the dipole moment spatial orientation factor, usually taking the average 2/3; *φ* is the quantum efficiency of PPE, usually a quantum efficiency of Try residues of 0.15; *n* is the refractive index of the medium, representing the average of water and organic matter; *J* is the overlap integral of the absorption spectra and the fluorescence spectra; and *ɛ*_A_*(λ)* is the molar extinction coefficient (34–36).

### 2.7 Ultraviolet–visible (UV–vis) spectroscopy absorbance measurements

The UV–vis absorption spectra of porcine pancreatic elastase PPE (2.5 mL) were recorded using a Cary 300 spectrometer (Agilent, San Diego, CA, United States) in a quartz cuvette with a path length of 10 mm. PPE concentration was fixed at 5 μM and the drug concentrations were 0 and 15 μM. Tris–HCl solution (pH 7.6) was used as the blank. The control group was a solution of four flavonoid monomers at a final concentration of 15 μM. The spectra were recorded in the range of 200–500 nm with a slit width of 2 nm at 298 K.

### 2.8 Synchronous fluorescence spectra measurements

For the synchronous fluorescence spectroscopy experiments, the configuration of the solution preparation was the same as that used in experiment 2.3. Synchronous fluorescence spectra of PPE-quercetin, PPE-hyperoside, PPE-luteolin, and PPE-luteoloside were recorded at 298 K. The appropriate excitation and emission wavelengths were set so that Δλ = 15 nm and 60 nm, respectively.

### 2.9 FT-IR spectra measurements

FTIR spectra were obtained using the potassium bromide (KBr) compression method with a Nicolet IS-50 spectrometer (Thermo Fisher Scientific, Waltham, MA, United States). In brief, 2 μL each of PPE, PPE-quercetin, PPE-hyperoside, PPE-luteolin, and PPE-luteoloside were individually applied to freshly prepared KBr compression sheets. The concentrations of PPE and the four isomers were 10 μM and 100 μM, respectively. The sheets were then dried at 45 °C and pressed. Blank background for the PBS buffer sheets. The spectral region between 4,000 and 500 cm^−1^ was selected to examine changes in the secondary structure resulting from enzyme-drug interactions. All samples were assayed after drying and the experimental conditions were consistent for each group of assays.

### 2.10 CD spectroscopy measurements

The CD spectra of PPE, PPE-quercetin, PPE-hyperoside, PPE-luteolin, and PPE-luteoloside were obtained using an MOS-500 CD spectrometer (Biologic Science Instruments, Seyssinet-Pariset, France). A quantity of PPE reserve solution, quercetin, hyperoside, luteolin and luteoloside reserve solution was pipetted with a pipette gun and placed in a 1 mm cuvette, at which point the five groups of samples were 5 μM PPE, 5 μM PPE mixed with 2.5 μM quercetin; 5 μM PPE mixed with 2.5 μM hyperoside; 5 μM PPE mixed with 2.5 μM luteolin; 5 μM PPE mixed solution with 2.5 μM luteoloside. That is, the concentration ratios of PPE to the five flavonoid monomers were 1:0 and 1:0.5, respectively, and the blank control group was a PBS buffer solution. Set at a temperature of 298 K. The signal was recorded from 190 to 260 nm using a path length of 1 mm, acquisition duration of 0.5 s, and scanning step of 2 nm. The CD spectra were averaged after performing three scans and correcting for the background value of the phosphate buffer. All results were documented as CD ellipticity in degrees.

### 2.11 Molecular docking analysis

AutoDock (4.2.6) docking software was used to explore the probable interactions between the four drugs and PPE. Repeat the simulation 3 times, 50 times per simulation. The grid point spacing was set to 4.00 Å, and the exhaustiveness was set to 100 to ensure a thorough search of the binding site. Protein structure data (PDB ID: 9EST) were obtained from the Protein Data Bank (http://www.rcsb.org/pdb/home/home.do). Three-dimensional (3D) structures of the four drugs were generated and optimized with the lowest energy using Chem Bio 3D Ultra 14.0, and then processed using Autodock 4.2.6, based on the addition of hydrogen atoms, calculation of electric charges, and docking with protein receptors.

### 2.12 Statistics and reproducibility

All the experiments were performed under the same conditions in triplicate, and the mean values were used for analysis. The software of Origin 2021 were used for the curves plotting and statistical processing.

## 3 Results

### 3.1 Evaluation of inhibition ability

In the process of novel drug discovery, drug activity screening methods are commonly employed. Selecting a suitable screening model can significantly reduce research expenditure and accelerate the overall experimental timeline. Models used for screening drug activity are generally divided into two main categories: *in vivo* systems and *in vitro* approaches. *In vivo* screening models are mainly used to test mammals and observe the therapeutic effects in animals. However, they are limited by their harsh requirements on the target, high cost, long experimental period, and other shortcomings. *In vitro* screening models are widely used by researchers because of their advantages that include low cost, fast experimental speed, and high efficiency. An *in vitro* enzyme target screening model was used to determine the inhibitory ability and type of inhibition of the drug on the enzyme. Crystallographic analyses have demonstrated that PPE and human leukocyte elastase (HLE) possess comparable structural characteristics. However, due to its greater availability and ease of extraction, PPE is frequently utilized as a representative model for investigating elastase-related enzymatic activity. PPE and HLE share high structural homology and a conserved catalytic triad, making PPE a well-established and reliable surrogate model for preliminary inhibitor screening; thus, the inhibitory effects observed here are expected to be predictive of HLE inhibition, although confirmation in future studies is warranted.

**Figure 2:**
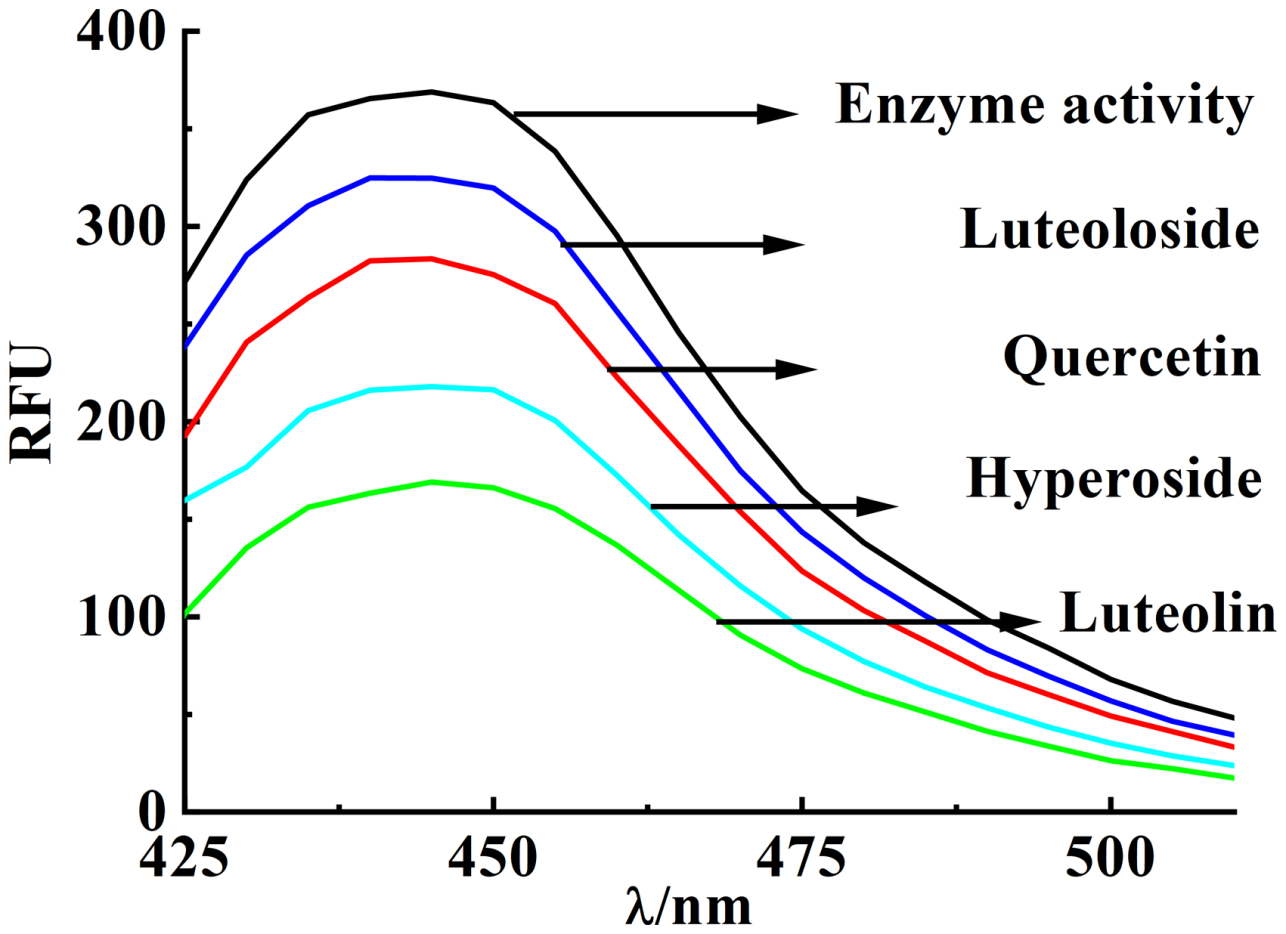
Inhibition rate of PPE by quercetin, hyperoside, luteolin and luteoloside under the same conditions.

In general, no fluorescence was observed at an excitation wavelength of 380 nm or an emission wavelength of 420–540 nm in the presence of PPE or fluorescent substrates. However, when the fluorescent substrates were decomposed using PPE, the maximum emission peak of the decomposition product was detected at 445 nm. Consequently, the rate of PPE inhibition by a drug can be characterized by its degree of fluorescence quenching. Although the structures of these four monomers are similar, their inhibitory abilities are substantially different. At the same concentration (22.5 μM), luteolin exhibited the strongest inhibitory effect, reaching 54.26% (Figure 2). The inhibitory effect of hyperoside was considerable (41.13%). The inhibitory effects of quercetin and luteoloside were relatively weak (22.18 and 12.15%, respectively).

**Figure 3:**
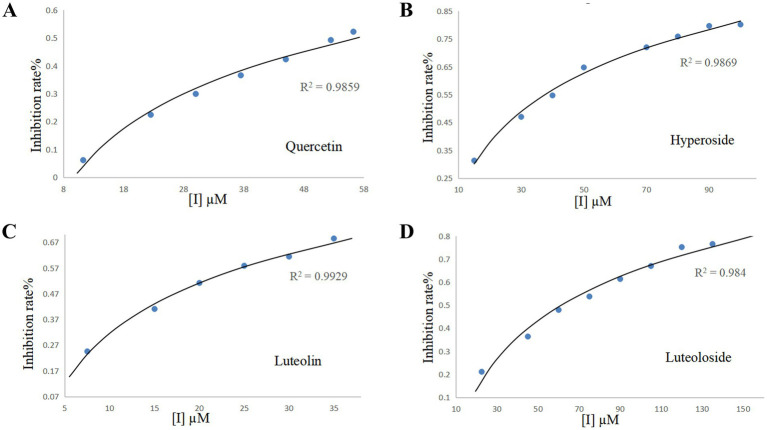
IC_50_ value of quercetin, hyperoside, luteolin and luteoloside **(A-D)** at 298K.

Quercetin, hyperoside, luteolin, and luteoloside inhibited elastase in dose-dependent manners under the appropriate concentration gradient (IC_50_ of 53.2, 31.01, 18.22, and 61.32 μM, respectively; Figure 3). Particular focus was placed on comparing the hydroxyl (OH) groups located at the C-3 position of the C-ring in quercetin and luteolin. This emphasis arose from the observation that the additional OH group present in the C-ring of quercetin significantly reduces its ability to inhibit elastase. The significant influence of O-glycosylation at the A-(C-7) and C-ring (C-3) positions was revealed by comparing the inhibitory effects of quercetin with those of hyperoside, luteolin, and luteoloside. Based on the IC_50_ values of quercetin and hyperoside, the 3-O-glycosylation of hyperoside increased its inhibitory activity, and the aglycones possessed stronger activity. In addition, 7-O-glycosylation of luteoloside reduces its activity. Glycosylation at position C-7 (the A-ring) has been suggested to produce steric hindrance that prevents molecules from binding to enzymes.

### 3.2 Fluorescence quenching mechanism of PPE by the four flavonoids

Fluorescence spectroscopy is often used to analyze the interaction between small molecule compounds and biological macromolecules. This method has the advantages of high sensitivity, simple and fast operation, need for fewer samples, high selectivity. Fluorescence spectroscopy can enable the understanding of the interaction between small molecules and biological macromolecules of the fluorescence burst mode, burst constants, energy transfer, binding constants, number of binding sites, type of force and formation of complexes, and other aspects (37). Endogenous fluorescent proteins generally contain three fluorescent aromatic amino acid residues: tryptophan (Trp), tyrosine (Tyr), and phenylalanine (Phe), with Phe having the lowest quantum yield. When the excitation wavelength is set to 280 nm, the fluorescence sources are mainly Trp and Tyr residues. When the excitation wavelength is set to 295 nm, the fluorescence source is Trp residues, although the intensity is relatively weak (38).

**Figure 4:**
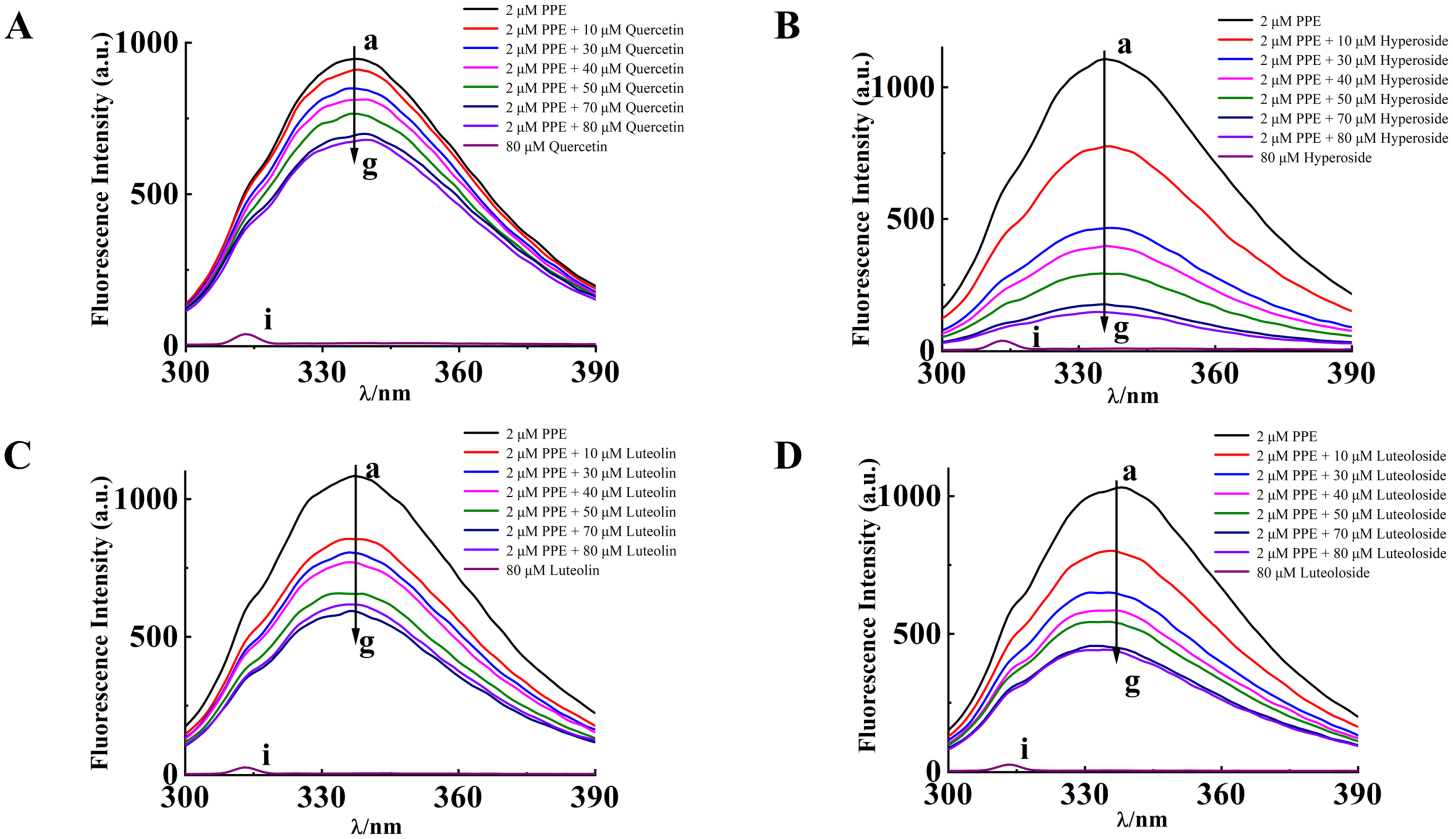
The intrinsic fluorescence spectra of PPE in the presence of quercetin **(A)**, hyperoside **(B)**, luteolin **(C)** and luteoloside **(D)** for curves a~g at T = 298 K, [PPE] = 2 μM, [quercetin, hyperoside, luteolin, luteoloside] = 0, 10, 30, 40, 50, 70, 80 μM, curve i denotes the emission spectra of quercetin, hyperoside, luteolin, luteoloside, [quercetin, hyperoside, luteolin, luteoloside] = 80 μM.

PPE exhibits intrinsic fluorescence in the presence of Trp, Tyr, and Phe residues. This fluorescence is primarily attributed to Trp because Tyr is highly unstable, whereas the quantum efficiency of Phe is extremely low (27, 39). Figure 4 shows the fluorescence spectra of PPE in the absence and presence of varying concentrations of the four monomers at 298 K. PPE exhibited the highest fluorescence emission at an excitation wavelength of 280 nm, with the corresponding emission peak observed at 336 nm. The fluorescence intensity of PPE decreased progressively without any significant peak shifts. An increase in the quercetin, hyperoside, luteolin, and luteoloside monomer contents resulted in a quenching effect on PPE.

Table 1 lists the *K_sv_* and *K_q_* values at 298, 303, and 310 K (Equation 2). The *K_sv_* values of PPE-quercetin, PPE-hyperoside, PPE-luteolin, and PPE-luteoloside decreased with increasing temperatures. *K_q_* values at the three temperatures exceeded 2.0 × 10^10^ L/(mol·s), indicating that the fluorescence quenching mode of PPE by the four monomers was static quenching.

**Table 1:** Quenching rate constants (Kq) and Stern-Volmer quenching constants (Ksv) at different temperatures.

System	*T/K*	*K_SV_/*10^4^ (L•mol^−1^)	*K_q_/*10^12^ (L•mol^−1^•s^−1^)	*r*
PPE-quercetin	298	0.59	0.59	0.99
	303	0.58	0.58	0.99
	310	0.52	0.52	0.98
PPE-hyperoside	298	5.47	5.47	0.99
	303	4.85	4.85	0.99
	310	4.78	4.78	0.99
PPE-luteolin	298	1.15	1.15	0.99
	303	1.04	1.04	0.98
	310	0.98	0.98	0.99
PPE-luteoloside	298	1.79	1.79	0.99
	303	1.66	1.66	0.99
	310	1.57	1.57	0.98

*r* denotes the Pearson correlation coefficient.

### 3.3 PPE binding parameters of the four flavonoids

#### 3.3.1 Binding constants and binding sites

The calculated *K_a_* and *n* values for log [1/([*Q_0_*]–[*P_0_*](*F_0_*-*F*)/*F_0_*)] and log [(*F_0_*-*F*)/*F*] are listed in Table 2. The value of n was close to 1, indicating that the drugs bound to PPE in 1:1 stoichiometry. The *K_a_* values of PPE-quercetin, PPE-hyperoside, PPE-luteolin, and PPE-luteoloside decreased with increasing temperature, further confirming the static quenching produced by these interactions.

**Table 2:** Binding constants (*K_a_*) and number of binding sites (*n*) for quercetin, hyperoside, luteolin, and luteoloside binding to PPE at various temperatures.

System	*T/K*	*K_a_/*10^4^ (L•mol^−1^)	*n*	*r*
PPE-quercetin	298	0.55	1.13	0.99
	303	0.53	0.99	0.99
	310	0.41	0.80	0.98
PPE-hyperoside	298	4.07	1.58	0.99
	303	3.38	1.56	0.99
	310	3.19	1.35	0.99
PPE-luteolin	298	1.21	0.97	0.99
	303	1.05	1.11	0.98
	310	0.98	1.17	0.99
PPE-luteoloside	298	1.91	0.95	0.99
	303	1.86	0.93	0.99
	310	1.81	1.07	0.98

#### 3.3.2 Thermodynamic parameters and types of forces

The thermodynamic parameters at 298, 303, and 310 K were calculated using the van’t Hoff thermodynamic formula: Table 3 presents the values of ΔH, ΔS, and ΔG for reference (Equations 4–6). According to the Ross theory, the main force is hydrophobic if Δ*H >* 0 and Δ*S >* 0, hydrogen interactions or van der Waals forces if Δ*H <* 0 and Δ*S <* 0, and electrostatic attraction if Δ*H <* 0 and Δ*S >* 0 (40–42). Specific to PPE-quercetin, PPE-hyperoside, PPE-luteolin, and PPE-luteoloside, ΔG < 0 indicated a spontaneous interaction between PPE and the four monomers. Thus, hydrogen interactions and van der Waals forces were the main interactions between PPE-hyperoside and PPE-luteolin, whereas electrostatic attraction played a major role in the interactions between PPE-quercetin and PPE-luteoloside. The strength of the interaction between hyperoside and luteolin exceeded that between quercetin and luteoloside. Therefore, the differences in the main forces may be one of the factors affecting the ability of the four monomers to inhibit PPE.

**Table 3:** Thermodynamic parameters for the interactions of quercetin, hyperoside, luteolin, and luteoloside with PPE.

System	*T/K*	Δ*H* kJ/mol	Δ*S* J/(mol•K)	Δ*G* kJ/mol
PPE-quercetin	298			−21.56
	303	−7.954	45.6521	−21.79
	310			−22.11
PPE-hyperoside	298			−28.74
	303	−27.511	−4.108	−28.76
	310			−28.79
PPE-luteolin	298			−27.20
	303	−25.241	−6.585	−27.24
	310			−27.28
PPE-luteoloside	298			−24.41
	303	−2.924	72.107	−24.77
	310			−25.28

#### 3.3.3 Energy transfer and binding distance

**Figure 5:**
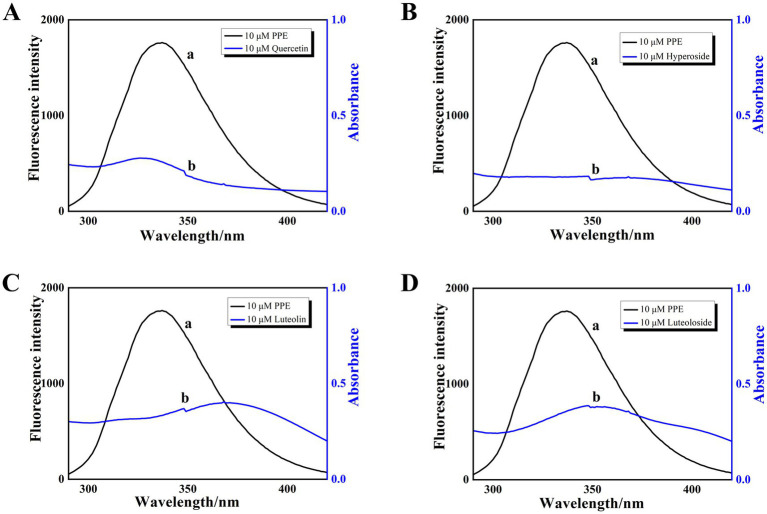
The overlap of fluorescence emission spectrum (curve a) and absorption spectrum (curve b) of PPE-quercetin **(A)**, PPE-hyperoside **(B)**, PPE-luteolin **(C)** and PPE-luteoloside **(D)**; [PPE] = [quercetin, hyperoside, luteolin and luteoloside] = 10 μM, T = 298 K.

The PPE-quercetin, PPE-hyperoside, PPE-luteolin, and PPE-luteoloside binding distances were calculated according to the fluorescence resonance energy transfer theory (Equations 7–9). For PPE-quercetin, PPE-hyperoside, PPE-luteolin, and PPE-luteoloside, the respective distance *r* value was 2.82, 2.40, 2.67 and 2.71 nm, respectively, and the respective *R_0_* value was 1.91, 1.86, 1.97 and 1.99 nm (Figure 5). It is possible that nonradiative energy transfer from PPE to quercetin, hyperoside, luteolin, or luteoloside occurred because the values of *r* were all < 7 nm, confirming the nonradiative energy conversion generated in the static quenching processes. Furthermore, the measured distance between the donor and acceptor was within the range of 2 to 8 nm, suggesting static quenching (43, 44).

### 3.4 Conformational changes of PPE induced by the flavonoids

#### 3.4.1 UV–vis absorption spectra

UV–vis spectroscopy is an important tool for analyzing structural changes in macromolecules in biomolecule-ligand binding studies. The characteristic peaks of the UV–vis spectra of proteins generally appear at 200 and 274 nm, which are due to the C=O jump in the polypeptide backbone and the absorption of Trp, Tyr, and Phe residues. Therefore, changes in the spectra before and after the addition of drug monomers can be observed to determine whether the secondary structure of the protein has changed. If the structure is changed, the UV absorption intensity changes or the position of the absorption peak shifts. In addition, the binding distance between the protein and drug monomer can be calculated using UV absorption and fluorescence values (45).

**Figure 6:**
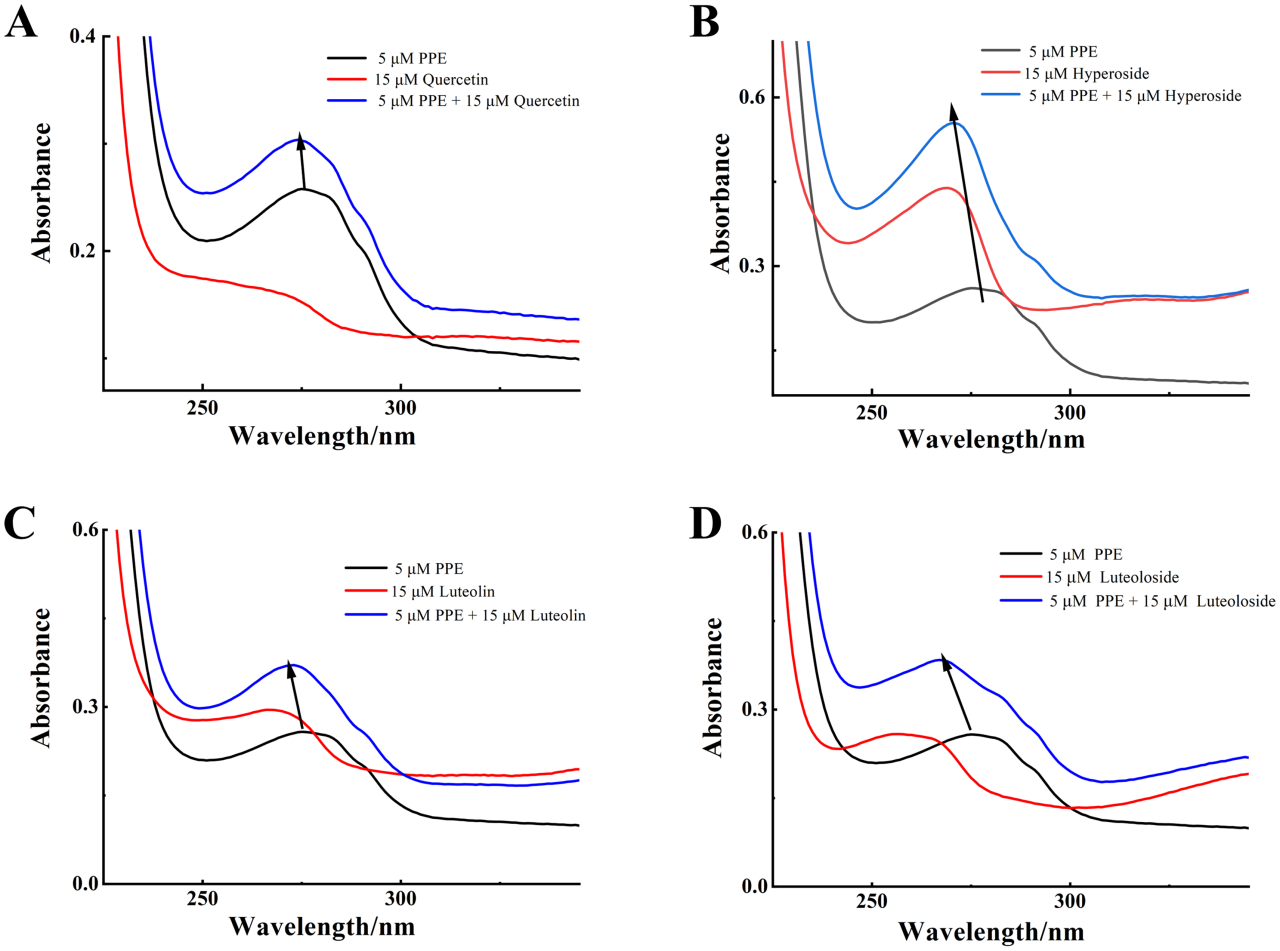
UV-vis absorption spectra of the PPE-quercetin **(A)**, PPE-hyperoside **(B)**, PPE-luteolin **(C)** and PPE-luteoloside **(D)** at 298K, pH 7.4; [PPE] = 5 μM, [quercetin, hyperoside, luteolin and luteoloside] = 15 μM.

UV–vis absorption spectroscopy was used to record the absorption of the PPE chromophores in the presence and absence of the four monomeric compounds. As illustrated in Figure 6, PPE displayed a maximum absorption peak at 275 nm before the separate addition of each monomer solution. This finding corresponds to the absorption of the aromatic residues, Try, Tyr, and Phe (46, 47). After separate addition of each flavonoid at the same concentration, the maximum absorption peaks of PPE underwent blue shifts of 2, 4, 3, and 8 nm for quercetin, hyperoside, luteolin, and luteoloside, respectively. The absorbance corresponding to the maximum absorption peak of PPE increased significantly. At 275 nm, A_PPE_ = 0.2578, A_Quercetin_ = 0.1523, A_Hyperoside_ = 0.3947, A_luteolin_ = 0.2764, and A_Luteoloside_ = 0.1843; all A_PPE_ + A_monomer_ > A_PPE-monomer_. The absorbance of the sum of the two components was higher than that of the complexes, confirming interactions between the monomers and PPE. This shows that the drugs changed the secondary structure of PPE. Because the absorbance of dynamically quenched fluorescent substances is generally not affected by the quenching agent, the absorption spectrum of static quenching changes owing to changes in the ground-state molecules, which further proves that static quenching occurs.

#### 3.4.2 Synchronous fluorescence spectra analyses

For UV–vis absorption or fluorescence spectroscopy alone, synchronous fluorescence spectra can be used because the absorption or emission of Tyr and Trp overlap, making it difficult to distinguish them. By fixing the interval Δλ between excitation and emission wavelengths to 15 and 60 nm, respectively, structural information about Tyr and Trp residues can be provided, respectively. The high sensitivity, low interference and small spectral overlap of this method have driven its popularity (48).

**Figure 7:**
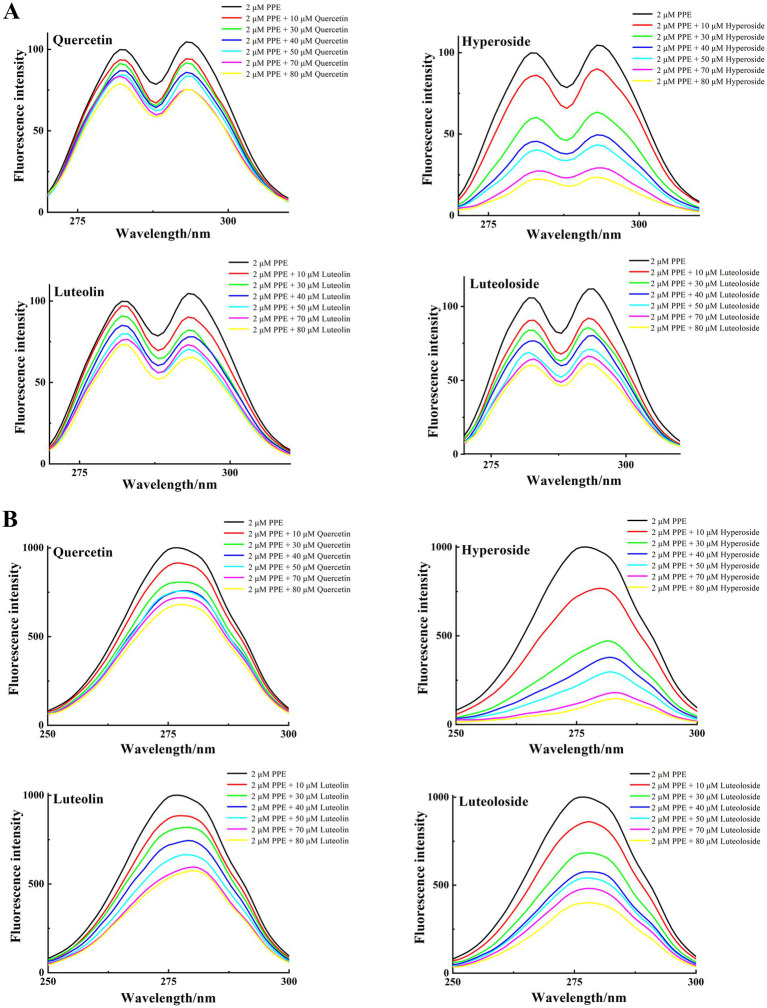
The synchronous fluorescence spectra of PPE in the absence and presence of quercetin, hyperoside, luteolin and luteoloside. **(A)** Δλ = 15 nm; **(B)** Δλ = 60 nm; at T = 298 K, pH 7.4. [PPE] = 2 μM, [quercetin, hyperoside, luteolin and luteoloside] = 0, 10, 30, 40, 50, 70, 80 μM.

Synchronous fluorescence studies of the microenvironmental changes around Tyr and Trp residues after binding of the four monomers to PPE were performed as previously described (49). Figure 7 shows the synchronous fluorescence spectra of Tyr (*Δλ*_ex-em_ = 15 nm) and Trp (*Δλ*_ex-em_ = 60 nm) in PPE after addition of various concentrations of quercetin, hyperoside, luteolin, and luteoloside. The fluorescence intensity of the Tyr residues decreased. However, none of them displayed significant peak shifts, indicating that the four monomers altered the Tyr conformation only slightly. However, the obvious effects of Trp were evident for all four monomers. Hyperoside and luteolin red-shifted by 7 and 4 nm, respectively, whereas quercetin and luteoloside red-shifted by 2 and 1 nm, respectively. Because the contribution of Tyr residues to PPE fluorescence, quenching was notably lower than that of Trp residues. All four monomers bound mainly to Trp residues in PPE. Based on these results, it can be concluded that all four compounds increased the polarity and hydrophilicity of the Trp residue microenvironment of PPE, weakened its hydrophobicity, increased the degree of extension of the peptide chain, and caused changes in the secondary structural conformation of PPE (50).

#### 3.4.3 FT-IR spectra measurements

In FT-IR spectroscopy, different functional groups exhibit different vibrational forms and absorption peaks. The FT-IR spectrograms of the enzyme before and after combination with the monomer of the TCM were used to judge the change in the secondary structure of the enzyme based on the change in the absorption peaks (51, 52).

**Figure 8:**
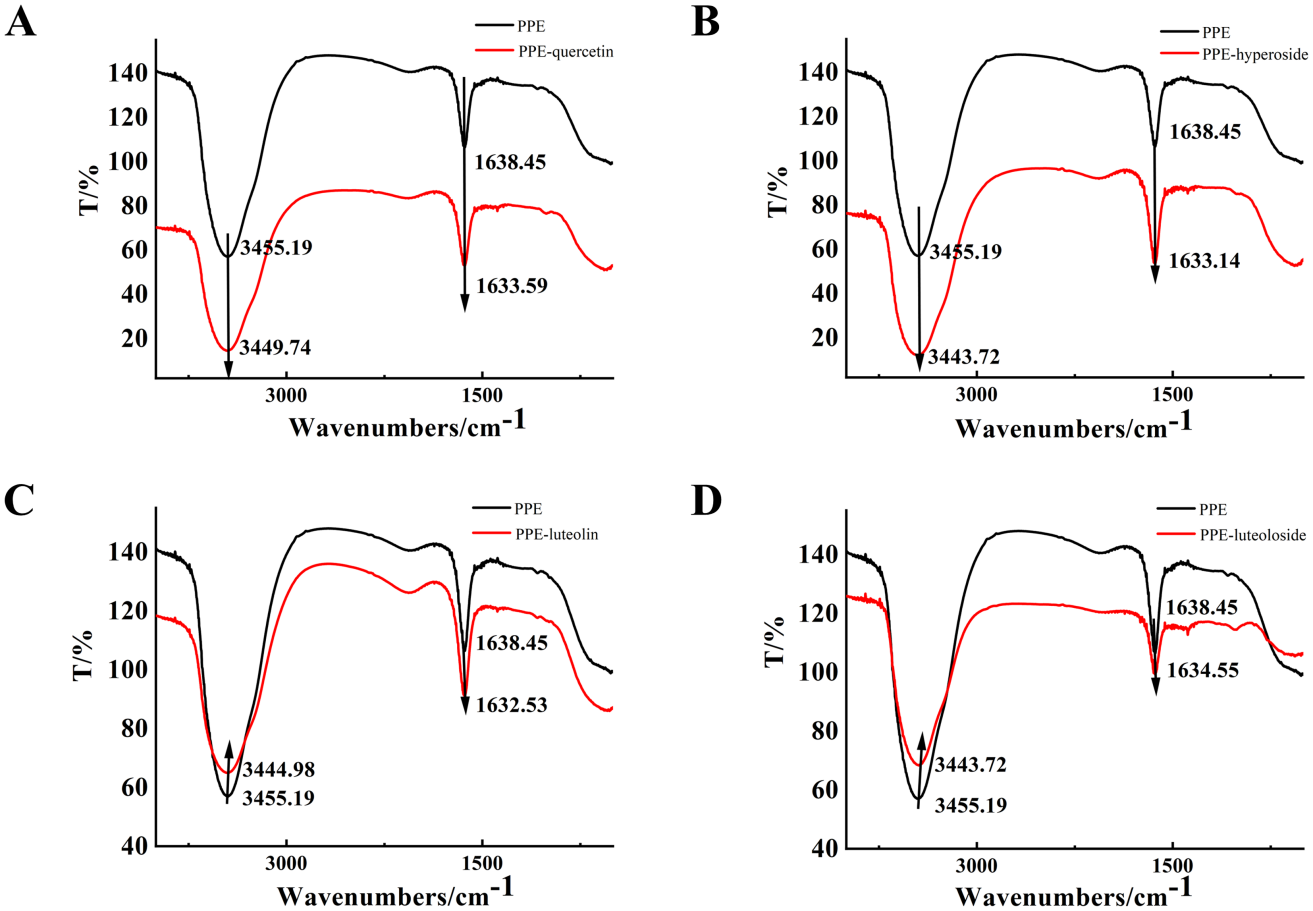
The FT-IR spectra of PPE-quercetin **(A)**, PPE-hyperoside **(B)**, PPE-luteolin **(C)** and PPE-luteoloside **(D)**, (curve a) the FT-IR spectra of PPE, (curve b) the FT-IR spectra of the PPE-monomer complex, [PPE] = 10 μM [quercetin, hyperoside, luteolin and luteoloside] = 100 µM.

In the FT-IR spectrum, 3,455 cm^−1^ represents the stretching vibration of protein N-H, and 1,700–1,600 cm^−1^ represents the stretching vibration of the C=O bond of the amide I band (Figure 8). The hydrogen bonds formed between the carbonyl and amino groups are closely related to secondary structure, and the amide I band is highly sensitive to changes in the secondary structure of the protein. Therefore, changes in the amide I band are often used to characterize changes in the secondary structure (53). With the addition of quercetin, hyperoside, luteolin, and luteoloside, the amide I band of PPE moved from 1638.45 to 1633.59, 1633.14, 1632.53, and 1634.55 cm^−1^, respectively. This was due to the interaction of the drug with the C=O in PPE, and the electron cloud density of C=O decreased, causing the absorption peak to move toward a lower wavenumber. The FT-IR spectra showed that all four monomers changed the secondary structure of PPE. In particular, luteolin, which had the strongest inhibitory effect, showed the most obvious change, whereas the weakest, luteoloside, showed the smallest change, which supports the conclusion of the experiment described in Section 3.2.

#### 3.4.4 CD spectrum analysis

CD spectroscopy is a technique commonly used to examine the conformation of proteins (53). Biological macromolecules are circular and dichroic, therefore, changes in the secondary structure and conformation of enzymes can be detected using CD, which has the advantages of low dosage, sensitivity, and rapidity. In general, the wavelengths of the α-helix poles usually appear at 207–210 and 221–222 nm as two negative peaks, and the random coil often appears at 195–202 nm as a strong negative peak (54).

**Figure 9:**
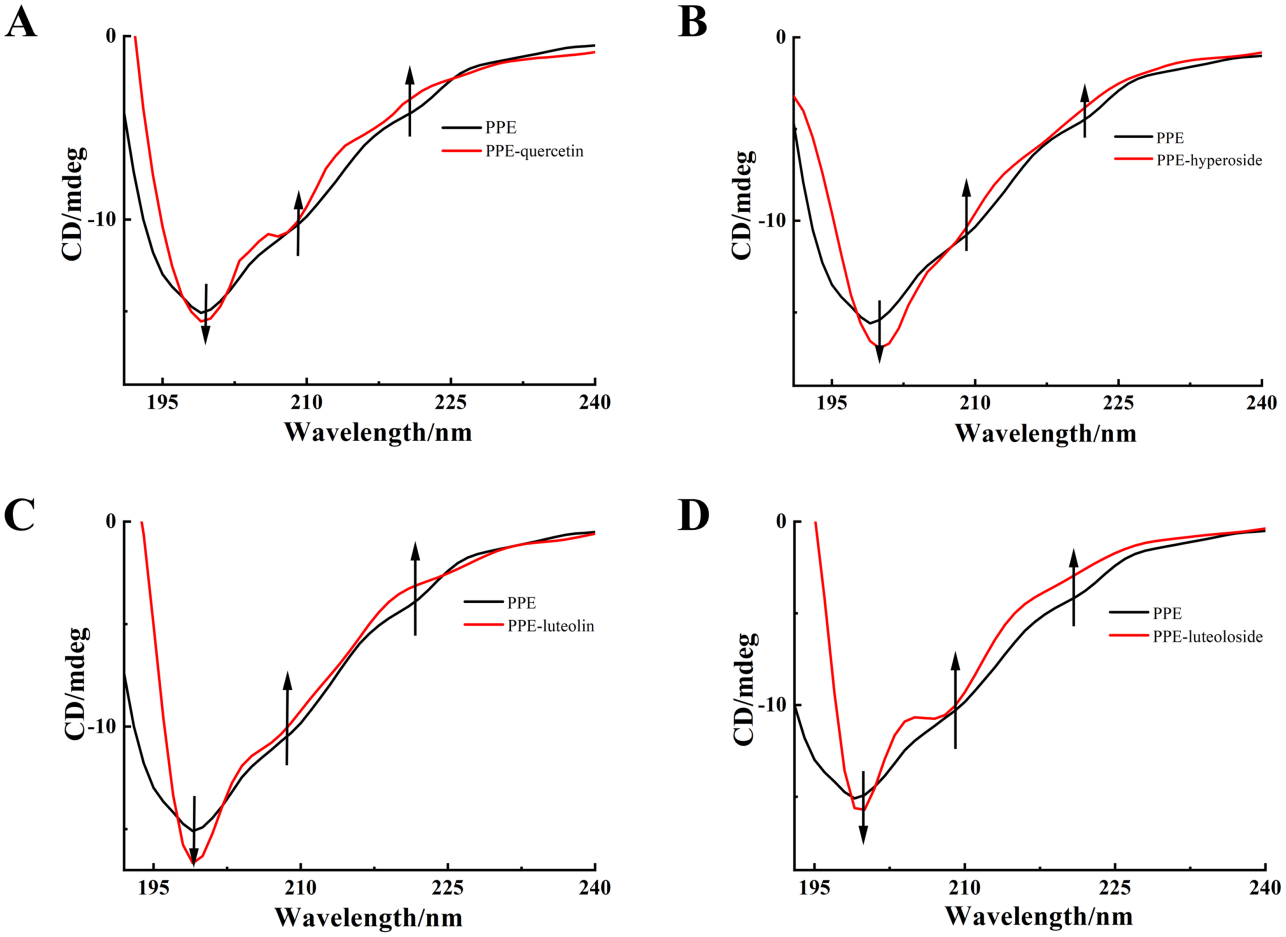
CD spectra of PPE, PPE-quercetin **(A)**, PPE-hyperoside **(B)**, PPE-luteolin **(C)** and PPE-luteoloside **(D)**, in PBS buffer at pH 6.8, T = 298 K. [PPE] = 5 μM; the mole ratio of quercetin/PPE, hyperoside/PPE, luteolin/PPE and luteoloside/PPE system were 0:1, 0.5:1, respectively.

To further study the influence of the four monomers on the conformation of PPE, the CD spectra of PPE and PPE-monomer systems were analyzed. Figure 9 shows the CD spectra of PPE in the absence and presence of the four monomers. In the four CD spectra, two negative bands were identified at wavelengths of 208 and 220 nm, indicative of the presence of the α-helical structure in PPE (55, 56). A strong negative peak was observed between 195 and 202 nm, which is the characteristic peak of the random coil of PPE (57). Upon the addition of four monomers at the same concentration, the α-helix content decreased and that of the random coil increased to varying degrees. The CD value was imported into the DicroProt software for calculations using the average of three determinations (Table 4). Luteolin and hyperoside, followed by quercetin and luteoloside, significantly increased the percentage of random coils. The α-helical structure of luteoloside, hyperoside, quercetin, and luteolin decreased by 1.99, 1.20, 1.14, and 0.96%, respectively. These findings suggest that the four monomers disrupt the bonding network by attaching to the primary polypeptide chains of the amino acid residues in PPE. The PPE structure becomes looser, part of the polypeptide chain expands, and the secondary structure changes (58). The random coil content increased in the following order: luteolin > hyperoside > quercetin > luteoloside, which was the same order as PPE inhibition. The four monomers also changed the microenvironment around PPE, increasing hydrophilicity and weakening hydrophobicity, consistent with the results of the fluorescence experiments.

**Table 4:** CD results for PPE, PPE-quercetin, PPE-hyperoside, PPE-luteolin, and PPE-luteoloside.

System	α-helix (%)	β-sheet (%)	Random coil (%)
PPE	7.50	51.50	41.00
PPE-quercetin	6.36	49.05	44.59
PPE-hyperoside	6.30	47.36	46.34
PPE-luteolin	6.54	46.53	46.53
PPE-luteoloside	5.51	50.58	43.91

#### 3.4.5 Molecular docking analysis

Molecular docking is a method for analyzing and modeling the geometrical configuration of molecules to perform intermolecular interactions and predict the structure of receptor–ligand complexes using methods such as chemometrics. Molecular docking has been widely used in anticancer drug and enzyme inhibitor screening. Owing to its unique advantages, it can improve the success rate of drug screening and reduce time and cost. Molecular docking can visualize the optimal binding site, type of force, amino acid information, and other relevant parameters of interactions between biomolecules and small drug molecules (59, 60).

**Figure 10:**
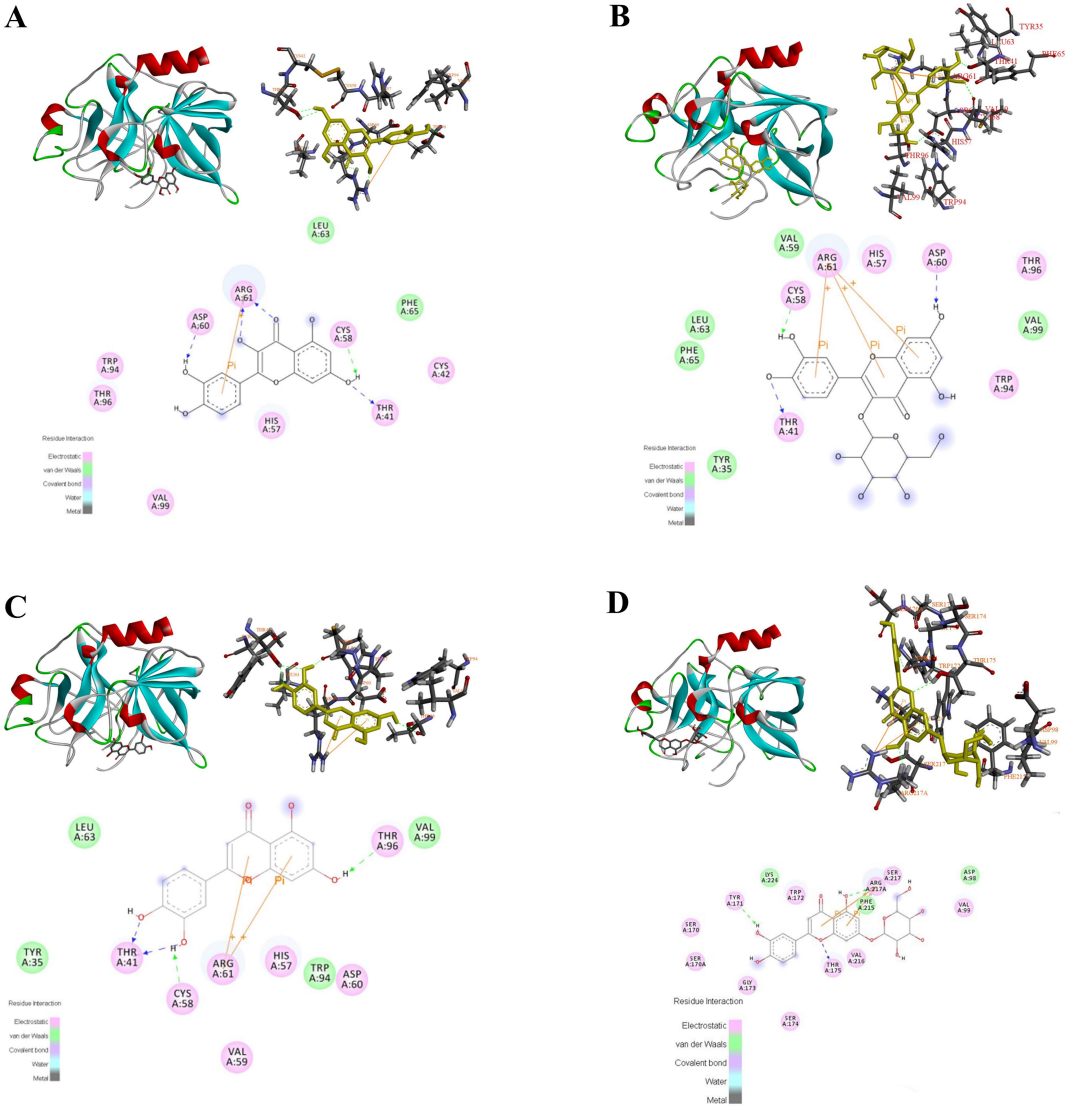
Molecular docking results of quercetin, hyperoside, luteolin and luteoloside **(A-D)** binding to PPE.

Molecular docking simulations were performed to determine the probable binding sites and forces for PPE-quercetin, PPE-hyperoside, PPE-luteolin, and PPE-luteoloside. The results of the docking analysis are presented in Figure 10 and Table 5. Hydrogen bonds, van der Waals forces, static electricity, and *π*-cation interactions were evident between the four monomers and PPE. The inhibition of PPE activity stabilizes the PPE-monomer complex through these forces. The main forces were electrostatic forces for quercetin and luteoloside, and hydrogen bonds and van der Waals forces for hyperoside and luteolin. At the same monomer concentration, quenching was the lowest for quercetin. This was because quercetin did not interact with the Trp and Tyr residues.

**Table 5:** Molecular docking results for the binding of quercetin, hyperoside, luteolin, and luteoloside to PPE.

Complexes	Amino acid residues	H-bond	Van der Waals	Electrostatic	π-Cation
PPE-quercetin	THR96, VAL96, ASP60, ARG61, HIS57, CYS58, LEU63, PHE65, CYS42, THR41	ASP60, ARG61 (2), CYS58, THR41	LEU63, PHE65	THR96, VAL96, ASP60, ARG61, HIS57, CYS58, CYS42, THR41	ARG61
PPE-hyperoside	LEU63, PHE65, TYR35, VAL59, CYS58, THR41, ARG61, HIS57, ASP60, THR96, VAL99, TRP94	CYS58, THR41, ASP60	LEU63, PHE65, TYR35, VAL59, VAL99	CYS58, THR41, ARG61, HIS57, ASP60, THR96, TRP94	ARG61 (3)
PPE-luteolin	TYR35, LEU63, THR41, CYS58, ARG61, VAL59, HIS57, TRP94, THR96, VAL99	THR41 (2), CYS58, THR96	TYR35, LEU63, TRP94, VAL99	THR41, CYS58, ARG61, VAL59, HIS57, THR96	ARG61 (2)
PPE-luteoloside	SER170, SER170A, TYR171, SER174, TRP172, THR175, ARG217A, SER217, VAL99, LYS224, PHE215, ASP98, GLY173	TYR171, THR175, ARG217A	LYS224, PHE215, ASP98	SER170, SER170A, TYR171, SER174, TRP172, THR175, ARG217A, SER217, GLY173	ARG217A (2)

PPE inhibition was stronger for luteolin than for quercetin. Combined with the results of molecular docking, luteolin A, B, and C rings were all closely bound to PPE. Quercetin is mainly composed of B and C rings, which may account for its weak inhibition. Compared with luteolin, 7-O-glucoside did not substantially interact with PPE. In contrast, steric hindrance increased, which may explain the weak inhibition of luteoloside. Although the 3-O-galactoside of hyperoside did not directly interact with PPE, it affected the A-, B-, and C- rings. The resulting interaction between ARG61 and the *π*-cation made the structure more stable. This was probably because the inhibitory activity of hyperoside was stronger than that of quercetin.

## 4 Discussion

This study provides a comprehensive evaluation of the inhibitory effects and interaction mechanisms of four structurally similar flavonoids—quercetin, hyperoside, luteolin, and luteoloside—on PPE. By employing multiple complementary techniques, including fluorescence quenching, thermodynamic analysis, UV–vis, FT-IR, CD spectroscopy, and molecular docking, we explored not only the inhibitory activity, but also the molecular basis of flavonoid–elastase interactions.

Of the four flavonoids, luteolin exhibited the strongest elastase inhibition, followed by hyperoside, quercetin, and luteoloside. The inhibitory capacity correlated with changes in the secondary structure of PPE, as demonstrated by CD and FT-IR spectroscopy, where increased random coil content and decreased α-helical content were observed. Fluorescence quenching and thermodynamic analyses revealed that static quenching was the dominant mode of interaction, with hydrogen bonding and van der Waals forces contributing primarily to hyperoside and luteolin binding. Electrostatic forces played a larger role in quercetin and luteoloside binding. These findings were further supported by molecular docking results, which provided insights into the spatial fit and interaction residues.

To benchmark the potency and mechanistic characteristics of the flavonoids studied, we performed a comparative analysis with sivelestat sodium, a clinically approved synthetic elastase inhibitor, previously investigated using similar spectroscopic and molecular docking methods (61). In our earlier study, sivelestat sodium exhibited strong elastase inhibition with an IC₅₀ value of 9.98 μM. Among the flavonoids tested in the present study, luteolin demonstrated the greatest potency, with a moderate inhibitory strength that approached that of sivelestat sodium.

Both luteolin and sivelestat sodium interacted with PPE through a static quenching mechanism and formed stable 1:1 complexes. Spectroscopic analyses revealed that both compounds induced similar conformational changes in PPE, including a decrease in α-helix content and an increase in random coil structure, as evidenced by CD and FT-IR spectra. Furthermore, both interactions are primarily stabilized by hydrogen bonding and van der Waals forces, indicating their mechanistic similarities. Although sivelestat sodium serves as an effective reference inhibitor, it is associated with relatively high cost and known side effects in clinical applications. In contrast, luteolin and related flavonoids have a natural origin, lower toxicity, and structural flexibility, which may enable future optimization. Although less potent than sivelestat, luteolin represents a promising natural scaffold with favorable safety and modifiable structure. These results highlight the therapeutic potential of flavonoids as natural alternatives to synthetic elastase inhibitors.

Compared with previous studies that often relied on crude plant extracts or focused solely on IC₅₀ values, our work provides a more integrated and mechanistic interpretation of flavonoid-induced inhibition of elastase. By employing a combination of spectroscopic analyses and molecular docking, we systematically examined how structural differences among four flavonoids (quercetin, hyperoside, luteolin, and luteoloside) influenced their inhibitory behavior and interaction with the enzyme.

Our data reveal a clear structure–activity relationship (SAR). 3-O-Glycosylation, as seen in hyperoside, enhanced elastase inhibition compared to its aglycone, quercetin, likely due to improved solubility or additional hydrogen bonding. In contrast, 7-O-glycosylation of luteoloside reduced its inhibitory potency relative to that of luteolin, possibly due to steric hindrance at the A-ring, which interferes with optimal binding. Furthermore, the absence of a C-3 hydroxyl group in luteolin appears to improve its inhibitory activity compared with that of quercetin, suggesting that the presence of this group may introduce unfavorable interactions or alter the conformation of the flavonoid core.

These findings highlight the critical role of hydroxylation and glycosylation positions in modulating enzyme inhibition and provide insights into the design of more potent flavonoid-based inhibitors. Although this SAR analysis was limited to natural compounds, our results establish a foundation for future studies involving semisynthetic derivatives or flavonoid analogs with systematic substitutions aimed at optimizing both potency and selectivity.

Despite these strengths, several limitations of this study must be acknowledged. First, all experiments were conducted *in vitro*. Therefore, the biological relevance of these findings in cellular and *in vivo* models remains to be validated. Second, the absence of crystallographic or nuclear magnetic resonance structural data limits the resolution of binding site confirmation. Third, although PPE is structurally similar to the human neutrophil elastase, species-specific differences may influence its translational relevance. Although fluorescence spectroscopy and molecular docking were used to evaluate the binding parameters, methods such as surface plasmon resonance or isothermal titration calorimetry can provide more precise kinetic and thermodynamic data. These techniques will be considered in future studies to strengthen our understanding of binding dynamics. Future work will also focus on *in vivo* validation, testing in disease models, and potential co-crystallization studies to verify flavonoid–elastase interactions at atomic resolution.

It should also be noted that only four flavonoids have been studied, which limits their structural diversity. While these compounds were chosen for their structural relevance and comparative potential, future studies will include additional flavonoid subclasses to further validate and expand the observed structure–activity relationships. Nevertheless, our findings provide a solid foundation for further investigations aimed at evaluating the biological relevance and therapeutic potential of flavonoid-mediated elastase inhibition. Future studies should focus on verifying these effects in appropriate biological systems to bridge the gap between molecular mechanisms and physiological outcomes.

Furthermore, this study was limited to evaluating elastase inhibition in an isolated enzymatic context. Although this approach provides valuable mechanistic insights, it does not account for potential off-target interactions or the involvement of other enzymes and pathways within a physiological setting. The inflammatory response is a complex, multifactorial process, and elastase is only one of the key mediators. Future studies will include broader screening for flavonoid selectivity across serine protease families and systems biology approaches, such as pathway enrichment analysis and molecular network modeling. These studies will help delineate the broader pharmacological relevance and specificity of these flavonoids in a more biologically complex environment.

In conclusion, this study advances the understanding of how natural flavonoids interact with elastase to modulate its structure. These results provide a strong foundation for the rational design and development of flavonoid-based elastase inhibitors for therapeutic use in inflammation-related diseases.

## 5 Conclusion

We investigated the inhibitory abilities and mechanisms of action of four structurally similar natural elastase inhibitors (quercetin, hyperoside, luteolin, and luteoloside). Luteolin exhibited the highest potency, followed by hyperoside, quercetin, and luteoloside. Various spectroscopic techniques and molecular docking simulations were employed to examine the interactions between PPE-quercetin, PPE-hyperoside, PPE-luteolin, and PPE-luteoloside. All four monomers statically quenched PPE fluorescence. The active cavities of PPE were successfully occupied by quercetin and luteoloside via electrostatic interactions. The primary interactions in PPE-hyperoside and PPE-luteolin were H-bonds and van der Waals forces. UV–vis, synchronous fluorescence, CD, and FT-IR spectral data indicated alterations in the secondary structure of PPE. These findings were supported by molecular docking results. These results provide structural insights into the modulation of enzymes by natural products, and support their potential as targeted regulators of biological macromolecules. Collectively, these data suggest that the elastase inhibitory activity of luteolin is negatively affected by 7-O-glycosylation (A-ring) and additional 3-OH (C-ring) groups, whereas 3-O-glycosylation of quercetin is beneficial.

Sivelestat sodium exhibited strong elastase inhibition through hydrogen bonding and van der Waals interactions. Luteolin exhibited the most potent activity among the flavonoids tested, with similar binding forces and structural effects on PPE. These findings suggest that luteolin may serve as a viable natural alternative to synthetic inhibitors, such as sivelestat sodium.

In this study, we propose an innovative strategy for the identification of elastase inhibitors by investigating individual flavonoid compounds rather than relying on complex crude extracts. Distinct from conventional screening methods, our approach integrates mechanistic analysis through the use of advanced tools such as fluorescence spectroscopy and molecular docking simulations. This enables a more accurate assessment of the pharmacological potential of flavonoids and facilitates the elucidation of structure–activity relationships. Ultimately, the findings support a more rational and selective pathway for the development of potent elastase-targeting therapeutics.

## Data Availability

The original contributions presented in the study are included in the article/supplementary material, further inquiries can be directed to the corresponding author.
